# Temporal trends in online searches related to COVID-19 vaccine safety: A digital infodemiology study

**DOI:** 10.34172/hpp.43117

**Published:** 2024-10-31

**Authors:** Akshaya Srikanth Bhagavathula, Theodoros Daglis, Vijay Kumar Chattu

**Affiliations:** ^1^Department of Public Health, College of Health and Human Sciences, North Dakota State University, Fargo, ND, USA; ^2^University of the Aegean, 841 00 Syros, Greece; Agricultural University of Athens, 118 55 Athens, Greece; Technical University of Crete, 731 00 Chania, Greece; ^3^Center for Global Health Research, Saveetha Medical College and Hospitals, Saveetha Institute of Medical and Technical Sciences (SIMATS), Saveetha University, Chennai, India; ^4^Department of OS & OT, Temerty Faculty of Medicine, University of Toronto, Toronto, ON M5G 1V7, Canada; ^5^Center for Evidence-based Research, Global Health Research and Innovations Canada Inc. (GHRIC), Toronto, Canada

**Keywords:** Adverse effects, Artificial intelligence, Big data, COVID-19 vaccines, Epidemiology, Health behavior, Human, Internet, Public health, United States

## Abstract

**Background:**

The rapid development of COVID-19 vaccines may have raised public concerns about their safety and side effects in the United States (US). This study aimed to assess trends in online searches related to the safety and side effects of COVID-19 vaccines in the US from 2021-2022.

**Methods:**

Google COVID-19 Vaccination Search Insights was used to analyze searches about COVID-19 vaccine safety and side effects in the US from January 4, 2021, to November 21, 2022 (98 weeks). Data were scaled from 0 (low interest) to 100 (high interest) as a fixed scaling factor called scaled normalized interest (SNI) to indicate relative search interest over time and by location. A joinpoint regression analysis was used to determine the search trends during the study period.

**Results:**

Analysis included 709 counties across 38 US states. Searches of COVID-19 vaccine safety and side effects peaked in April 2021 in the District of Columbia (SNI: 35.8), Massachusetts (29.7), New Hampshire (27.4), Connecticut (27.3), and Maine (26.7), then decreased significantly by an average monthly percentage change (AMPC) of -16.6% (95% CI -19.9 to -13.3) until July 2022. Overall AMPC from January 2021 to November 2022 was -8.9% (95% CI -16.2 to -0.9; P<0.001).

**Conclusion:**

Online searches related to COVID-19 vaccine safety and side effects decreased dramatically over time, supporting the utility of digital surveillance to track real-time vaccine safety concerns. This study provides insights into public interest in COVID-19 vaccine risks and can help monitor potential safety issues.

## Introduction

 The COVID-19 pandemic triggered an unprecedented global health emergency, necessitating swift scientific advancements, notably the development and mass distribution of effective vaccines.^1^ Despite the rapid development and proven efficacy of these vaccines, public concerns regarding their safety and potential side effects have dominated discourse and media narratives.^2-4^Understanding public perception and interest in vaccine safety is crucial for public health responses. Google COVID-19 Vaccine Search Insights (G-VSI) provides a unique opportunity to gain real-time insights into public interest regarding COVID-19 vaccine safety.

 Google Search data provides insights into public perception and interest in any topic. Google search volume can reflect the public’s level of concern about a particular topic and provide information on individuals’ concerns. Particularly in 2021, the highest search volume for “COVID-19 vaccine side effects” was recorded in the United States^5,6^ and India,^7^ with other countries showing a similar trend. Prior studies suggest high public interest and concern about the safety and side effects of COVID-19 vaccines.^8-12^

 G-VSI is a public web tool developed by Google Inc. to help health authorities understand public concerns and provide accurate information on COVID-19 vaccines.^13^ It can be used to improve immunization programs and analyze their potential impact. For example, data can be compared with COVID-19 vaccine trackers to identify regions where people may hesitate to get vaccinated due to safety concerns. People may be more receptive to vaccines in locations with lower vaccination rates, high search intent, and low side effect searches. G-VSI provides aggregated, anonymized weekly data on COVID-19 vaccine safety and side effects from Google searches at the state, city, and zip code levels.^13-15^ However, no studies have used this G-VSI big data to provide public health insights on COVID-19 vaccine safety and side effects in the US. Therefore, this study aimed to assess search trends related to the safety and side effects of COVID-19 vaccines in the US from 2021-2022, contributing novel insights to ensure vaccine acceptance and advance global public health goals.

## Methods

 This retrospective cross-sectional study utilized weekly data from January 2021 to November 2022 obtained from G-VSI related to the safety and side effects of COVID-19 vaccines.^13^

 G-VSI provides an open-source, aggregated, anonymous search dataset on COVID-19 vaccination interest, safety, and side effects. Data are presented as scaled normalized interest (SNI) from 0 (no interest) to 100 (high interest). The G-VSI methodology is detailed elsewhere.^13-15^

 Briefly, weekly SNI values were determined by counting search queries related to COVID-19 vaccine safety and side effects for a given week and region and calculating normalized interest as the proportion of all regional search queries related to COVID-19 vaccine safety and side effects. The maximum weekly normalized interest value at the national US level was identified and scaled to 100 as a fixed factor. All other normalized interest values were scaled using this fixed factor to produce SNI values across regions, categories, and time.

###  Data variables

 The data variables presented in the G-VSI include:

- *Search volume:* Searches related to COVID-19 vaccine safety and side effects presented as SNI values.
- *Geographic region:* SNI is provided at the county level (US) and divided into three sub-levels – state, county, and postal code – according to the 2018 US Census Bureau.

###  Data process

 Data collection was carried out on November 26, 2022. The US vaccination search insights comprise 1 048 576 variables, with data subdivided by SNI at the country, state, county, and postal code levels. The obtained data were stratified by the approval dates of the following COVID-19 vaccines by the US Food and Drug Administration (FDA):

- BNT-162b2 (Pfizer-BioNTech) – 01/04/2021
- mRNA-1273 (Moderna) - 01/04/2021
- Ad26.COV2.S (Johnson & Johnson) – 01/01/2021
- NVX-CoV2373 (Novavax) – 07/01/2022
- Bivalent booster vaccine (Pfizer-BioNTech/Moderna) – 08/21/2022

###  Statistical analysis

 Various analytical methods were employed to comprehensively overview COVID-19 vaccine safety and side effect search trends at the county level following the FDA authorization timeline. First, a bubble map visualized county-level search trends for COVID-19 vaccine safety and side effects following FDA authorizations during the study period from January 4, 2021, to November 21, 2022. The monthly mean summarized online searches across 38 states. Joinpoint regression analysis was performed for each state to analyze time trends in SNI data using the Joinpoint Regression Program (version 4.9.1.0) from the National Cancer Institute.^16^ This software identifies changes in temporal trends (“joinpoints”) using regression modeling to estimate the regression function between joinpoints.^17^ Analysis settings allowed up to three joinpoints. The monthly percentage changes (MPC) between trend change points were determined with 95% confidence intervals (CI).

## Results

**Figure 1:**
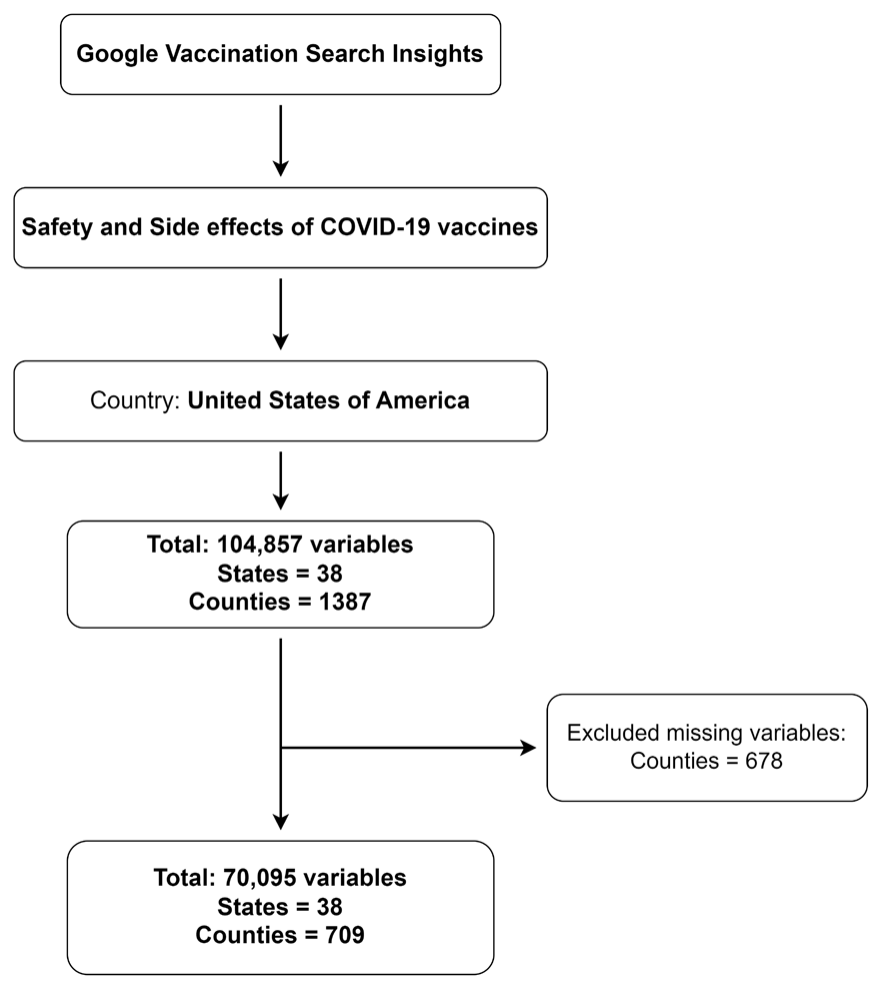
Flow of study sample

**Figure 2:**
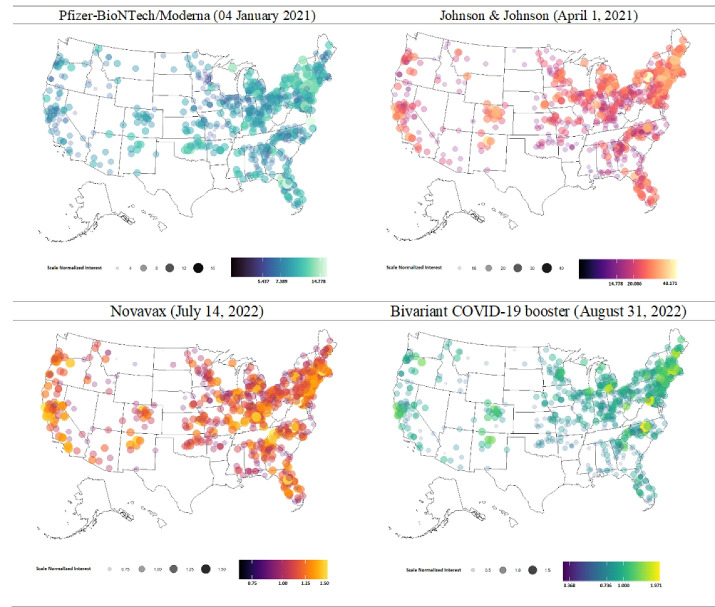
Search Insights related to COVID-19 vaccine safety and side effects of U.S. approved COVID-19 vaccines

 Analysis included 708 counties across 38 US states. After excluding missing values, 38 states and 709 counties were included in the final analysis. The detailed flow of included and excluded states and counties is shown in Figure 1. Analysis of G-VSI for COVID-19 vaccine safety and side effects across 708 counties showed an immediate spike in the first week of January 2021. The mean SNI was 8.5 ± 2.18, increasing to 21.7 ± 6.12 in April 2021. However, the number of searches steadily decreased over time, eventually falling below 1.5% (1.11 ± 0.18). Some counties in Northeast states saw SNI increases after the bivalent COVID-19 vaccine was approved. More details are provided in Figure 2.

Table 1 describes monthly searches related to COVID-19 vaccine safety and side effects in each US state from 2021-2022. Between January 4, 2021, and November 21, 2022, the highest mean SNIs were observed in the District of Columbia (6.3), Massachusetts (6.0), New Jersey (6.0), New York (6.0), and New Hampshire (5.8). The lowest mean SNIs were recorded in Mississippi (3.7), North Dakota (3.8), Louisiana (3.9), Oklahoma (4.1), and Arkansas (4.2).

 Joinpoint regression analysis was used to estimate MPC and explore monthly temporal trends in COVID-19 vaccine safety and side effect SNIs in the US during 2021-2022. Three models were fitted (Table 2). Overall, there was a significant decrease in SNI of -8.9% (95% CI: -16.2 to -0.9), with a specific decrease of -16.6% (*P* < 0.001) from April 2021 to July 2022. Between January and April 2021, states including the District of Columbia (MPC: 54.5%, 95% CI: 12–113), Maryland (54.5%, 95% CI: 12–113), Massachusetts (47.5%, 95% CI: 10.1-97.7), Colorado (47.4%, 95% CI: 13.3–91.7), Minnesota (44.4%, 95% CI: 10.5–88.6), New Hampshire (42.5%, 95% CI: 3.3–96.6), and Michigan (41.6%, 95% CI: 7.4–86.6) showed a significant spike in SNI. This sharp increase was followed by a significant decrease from April 2021 to July 2022.

**Table 1:**
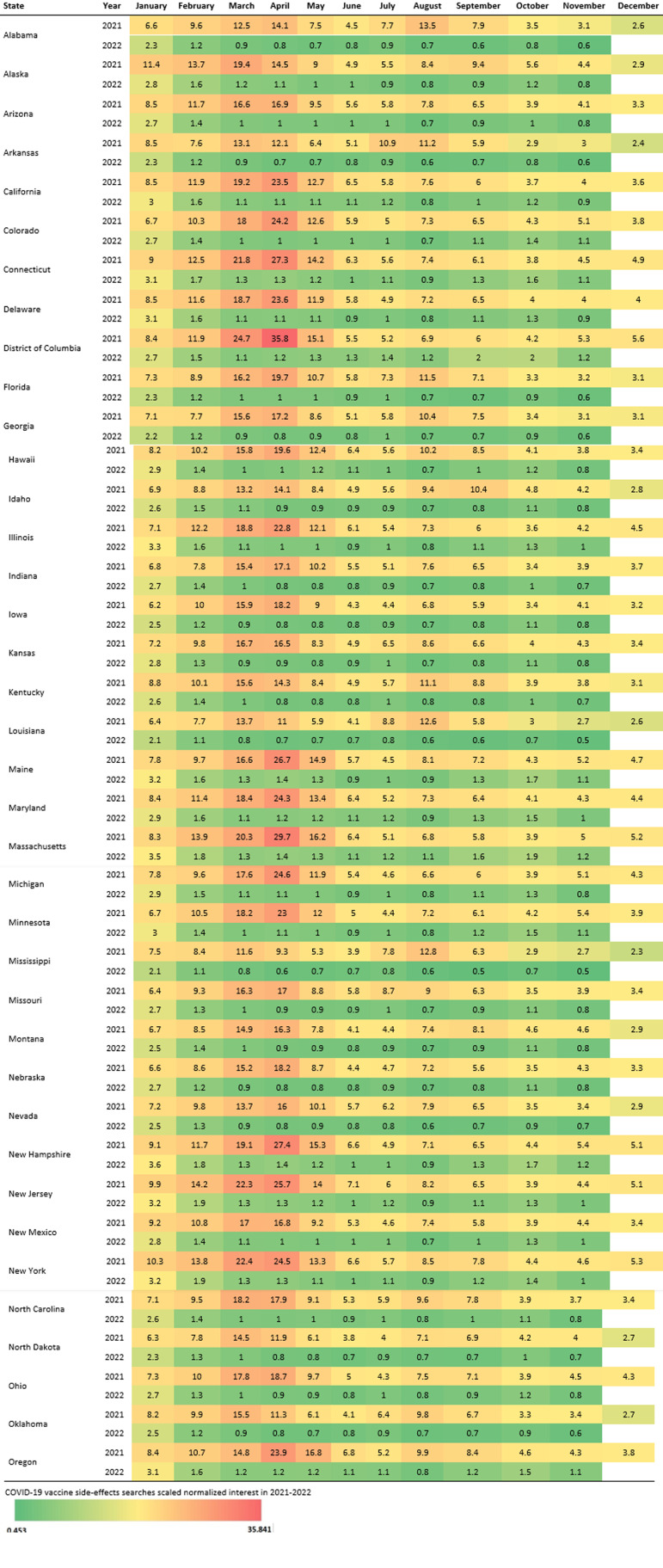
U.S. state-level online searches related to COVID-19 vaccine safety and side-effects from 2021–2022

**Table 2:** Trends in COVID-19 vaccine safety and side-effects online searches in the US

**States**	**January – April 2021**	**April 2021 – July 2022**	**July 2022 – November 2022**	**AMPC (95% CI)**
Overall	24.9 (-25.7 – 109.8)	-16.6* (-19.9 – -13.3)	0.5 (-12.9 – 15.9)	-8.9* (-16.2 – -0.9)
Alabama	0.4 (-10.4 – 12.3)	-15.5* (-21.0 – -8.9)	-4.0 (-12.4 – 5.3)	-11.2* (-16.6 – -5.5)
Alaska	6.0 (-20.0 – 40.6)	-15.8* (-23.8 – -7.2)	-3.4 (-7.9 – 1.3)	-10.9* (-18.2 – -2.9)
Arizona	17.5 (-26.4 – 87.5)	-16.6* (-19.5 – -13.6)	-1.6 (-13.5 – 11.9)	-9.7 (-19.7 – 1.5)
Arkansas	-1.3 (-11.2 – 9.7)	-15.1* (-20.5 – -9.2)	-3.2 (-11.2 – 5.6)	-11.2* (-16.3 – -5.8)
California	24.3 (-21.0 – 95.6)	-16.4* (-19.4 – -13.2)	-1.9 (-11.1 – 8.3)	-9.1* (-15.4 – -2.4)
Colorado	47.4* (13.3 – 91.7)	-18.6* (-28.3 – -7.7)	1.4 (-4.2 – 7.4)	-8.1* (-16.2 – 0.7)
Connecticut	23.9 (-28.7 – 115.2)	-16.4* (-19.9 – -12.8)	2.8 (-11.7 – 19.7)	-8.5* (-16.0 – -0.2)
Delaware	20.7 (-28.9 – 104.9)	-17.0* (-20.0 – -13.9)	5.0 (-13.4 – 27.4)	-8.9* (-16.0 – -1.2)
District of Columbia	54.5* (12.0 – 113.0)	-18.9* (-31.5 – -4.0)	5.6 (-1.6 – 13.2)	-7.1 (-17.8 – 5.1)
Florida	31.2 (-13.7 – 99.6)	-18.1* (-21.8 – -14.2)	-4.8 (-14.9 – 6.5)	-10.2* (-16.1 – -3.9)
Georgia	29.8 (-26.1 – 128.0)	-16.9* (-20.6 – -13.0)	-4.5 (-15.5 – 8.0)	-9.4 (-17.4 – -0.5)
Hawaii	26.7 (-20.2 – 100.9)	-17.2* (-21.4 – -12.9)	-2.8 (-14.1 – 9.9)	-9.7* (-16.2 – -2.7)
Idaho	18.5 (-9.1 – 54.4)	-15.3* (-22.7 – -7.1)	-2.3 (-6.5 – 2.2)	-8.9* (-15.8 – -1.3)
Illinois	28.8 (-25.1 – 121.5)	-16.7* (-20.1 – -13.2)	2.0 (-12.1 – 18.5)	-8.3* (-15.7 – -0.3)
Indiana	30.4 (-22.1 – 118.2)	-16.8* (-20.0 – -13.5)	-0.6 (-13.8 – 14.5)	-8.6* (-19.5 – 3.8)
Iowa	26.6 (-29.4 – 126.8)	-16.7* (-20.3 – -12.9)	1.9 (-13.2 – 19.7)	-8.3 (-19.5 – 4.5)
Kansas	22.9 (-21.2 – 91.7)	-16.4* (-28.2 – -2.7)	0.8 (-8.0 – 10.5)	-9.2 (-20.2 – 3.2)
Kentucky	-5.6 (-14.1 – 3.8)	-15.0* (-20.9 – -8.6)	0.2 (-10.8 – 12.5)	-11.2* (-16.4 – -5.6)
Louisiana	0.1 (-10.8 – 12.3)	-15.1* (-21.0 – -8.7)	-4.6 (-13.1 – 4.9)	-11.2* (-17.1 – -4.9)
Maine	28.7 (-32.3 – 144.8)	-16.5* (-20.1 – -12.7)	6.9 (-15.9 – 35.2)	-8.0 (-19.3 – 4.9)
Maryland	38.1* (7.7 – 77.0)	-18.0 (-28.0 – -6.5)	0.8 (-4.6 – 6.4)	-8.6 (-16.9 – 0.5)
Massachusetts	47.5* (10.1 – 97.7)	-18.5* (-30.1 – -5.0)	1.9 (-4.4 – 8.6)	-8.0 (-17.7 – 2.9)
Michigan	41.6* (7.4 – 86.6)	-18.6* (-29.6 – -5.9)	-0.2 (-6.1 – 6.0)	-8.9 (-18.0 – 1.2)
Minnesota	44.4* (10.5 – 88.6)	-18.3* (-28.1 – -7.1)	1.6 (-4.2 – 7.7)	-7.3 (-16.0 – 2.3)
Mississippi	-0.8 (-10.7 – 10.1)	-15.3* (-20.7 – -9.6)	-5.4 (-13.1 – 3.1)	-11.7* (-16.6 – -6.5)
Missouri	26.6 (-17.8 – 95.0)	-16.3* (-26.9 – -4.0)	-0.8 (-8.2 – 7.4)	-8.6 (-18.6 – 2.5)
Montana	22.6 (-13.3 – 73.4)	-15.9* (-25.3 – -5.4)	1.1 (-5.9 – 8.6)	-8.2 (-17.6 – 2.2)
Nebraska	26.4 (-30.4 – 129.6)	-16.6* (-20.3 – -12.7)	0.9 (-14.4 – 18.9)	-8.3 (-19.1 – 3.9)
Nevada	24.9 (-10.8 – 74.9)	-17.7* (-20.7 – -14.5)	-4.5 (-12.7 – 4.5)	-10.1* (-18.8 – -0.4)
New Hampshire	42.5* (3.3 – 96.6)	-18.5* (-31.1 – -3.4)	0.6 (-6.2 – 7.9)	-8.6 (-18.8 – 2.9)
New Jersey	20.3 (-26.3 – 96.3)	-16.7* (-19.8 – -13.6)	-0.4 (-12.9 – 14.0)	-9.6* (-16.2 – -2.4)
New Mexico	13.4 (-32.6 – 90.8)	-15.8* (-19.0 – -12.4)	1.3 (-12.2 – 17.0)	-9.1 (-20.3 – 3.7)
New York	19.0 (-30.7 – 104.3)	-17.3* (-20.3 – -14.1)	4.6 (-14.1 – 27.4)	-9.3* (-17.0 – -0.8)
North Carolina	27.7 (-8.4 – 78.1)	-16.2* (-25.3 – -5.9)	-2.1 (-7.5 – 3.5)	-8.6* (-17.5 – 1.2)
North Dakota	18.9 (-11.8 – 60.2)	-15.4* (-23.6 – -6.4)	0.2 (-5.8 – 6.6)	-8.3 (-16.0 – 0.1)
Ohio	26.8 (-19.4 – 99.5)	-16.5* (-28.6 – -2.3)	0.2 (-7.2 – 8.2)	-8.5 (-20.5 – 5.2)
Oklahoma	-6.8 (-15.5 – 2.7)	-15.0* (-21.2 – -8.4)	-0.9 (-12.1 – 11.7)	-10.3 (-19.8 – 0.4)
Oregon	29.4 (-16.4 – 99.8)	-17.2* (-20.8 – -13.4)	3.3 (-10.8 – 19.6)	-8.4* (-14.7 – -1.6)

AMPC: Average monthly percentage changes *significant at *P* < 0.05.

## Discussion

 This exploratory study that leveraged Google’s proprietary COVID-19 Vaccination Search Insights tool to analyze COVID-19 vaccine safety search trends in the US from 2021-2022. The findings reveal a spike in searches during the early vaccine rollout in 2021, likely reflecting public interest and concern about the rapidly developed new vaccines.^18,19^ The Northeast region showed the highest search levels, contrasting with surveys finding more hesitancy in the South and Midwest.^20,21^ This highlights a potential disconnect between online behavior and attitudes.^22^The subsequent marked decrease in searches over 2022 could signal reduced concern as people gained confidence in vaccine safety,^23^ however, it may also reflect a saturation of information needs.

 Ongoing monitoring is required, especially with new vaccine formulations. A French study by Ward et al. highlighted that mandated vaccination programs could increase immunization rates but do not address every problem affecting uptake. The authors stressed that any COVID-19 vaccination policy should be built around outreach initiatives and persistent efforts to encourage individuals who are hesitant.^24^ Besides, the COVID-19 vaccine campaigns were at risk of vaccine hesitancy and politicization, as Peretti-Watel reported in their research in France. They found that the willingness of participants to accept a SARS-CoV-2 vaccine was significantly influenced by their choice of candidate in the first round of the 2017 presidential election, with voters who supported candidates on the far left or far right significantly more likely to reject the vaccine.^25^

 The shifting search trends underscore evolving public sentiment about the COVID-19 vaccines. Heightened interest and worry early on led to declining searches over time as immunization expanded.^26^ A US study highlighted that political polarization and the spread of vaccine skepticism by conservative media and political figures created anti-vaccine views among a significant portion of the population in some parts of the US.^27^ Another study from the United Kingdom concluded that coordinated efforts by physicians, policymakers, health authorities, and vaccine manufacturers at the national and grassroots levels are critical to meeting COVID-19 immunization targets.^28^ These findings demonstrate the value of digital surveillance, particularly search data, for real-time tracking of public perspectives. Monitoring searches can aid in rapidly identifying and responding to emerging safety issues ahead of traditional reporting systems.

 Leveraging real-time search trends allows public health authorities to swiftly detect signals around vaccine hesitancy and concerns.^29^ Targeting communication to regions with more persistent safety questions could further improve vaccine acceptance.^30^ Mello et al reported that public communication of studies demonstrating vaccine safety has been suboptimal, with media emphasizing vaccines’ association with specific adverse events rather than their overall beneficial benefit-to-risk ratio. These issues may decrease compliance with COVID-19 immunization obligations without a coordinated and effective public education campaign.^31^ Therefore, it is essential to have transparent communications to avoid vaccines becoming a part of political debate.^25^

 Monitoring search trends provides a supplementary data stream for identifying and responding to emerging safety issues faster than traditional reporting.^32^ Another US study by Mello et al. on the effectiveness of vaccine mandates in improving the uptake of COVID-19 vaccines highlighted that effective vaccination programs require diligent observation of adverse events following immunization, as well as clear, sophisticated communication of findings to the public.^31^ This enables data-driven communication efforts to address questions and further improve vaccine acceptance.^30,32,33^ However, there is also a differential adoption of COVID-19 vaccines in the US states, which plays a significant role in vaccine acceptance. As highlighted in the National Academy for State Health Policy document, except for entities subject to federal jurisdiction, COVID-19 vaccine obligations are the responsibility of states, municipalities, and corporations. Mandatory adoption will vary across the country due to ideological differences. Areas with the lowest immunization rates are less likely to mandate vaccination. Some states have passed laws restricting some or all COVID-19 vaccine mandates.^34^

 This study has limitations, including assessing aggregate search trends but not individual-level motivations. Additionally, Google users represent a subset of the population, so the results may not be generalized.^35^ Future studies could survey searchers on reasons for seeking vaccine safety information online to clarify interpretations. Other limitations are the lack of demographic data and reliance on a proprietary algorithmic tool. However, this exploratory study supports the value of digital trace data for gauging population perspectives on vaccines.

 In conclusion, this exploratory infodemiology study analyzed Google search trends to gauge public interest in COVID-19 vaccine safety from 2021-2022. Findings reveal heightened online searches about vaccine side effects during the initial rollout, followed by a marked decline over time as immunization expanded. These trends likely reflect uncertainty early on, transitioning to greater confidence as real-world evidence on safety accumulated. However, integrating digital surveillance with other data streams is crucial for rapidly detecting emerging issues and guiding communication strategies that proactively address hesitancy. Overall, this research demonstrates the potential value of unobtrusively mining search query data to monitor real-time population perspectives on novel vaccines. With appropriate privacy diligence, these rapid insights can complement traditional pharmacovigilance to promote vaccine uptake, safety, and effectiveness during public health emergencies.

## Competing Interests

 None to declare.

## Data Availability Statement

 The data supporting this research are accessible at https://google-research.github.io/vaccination-search-insights/.html.

## Ethical Approval

 Not applicable.
